# Supplementary material for: Comparing psychological versus pharmacological treatment in emotional disorders: A network analysis
Source: PLoS One. 2024 Apr 3;19(4):e0301675. doi: 10.1371/journal.pone.0301675 (PMC10990220; doi:10.1371/journal.pone.0301675)
Supplement: S2 Fig — (PDF) [file pone.0301675.s002.pdf]

Supplementary Figure S2. Bootstrapped sampling distribution of edge weight estimates at post-treatment

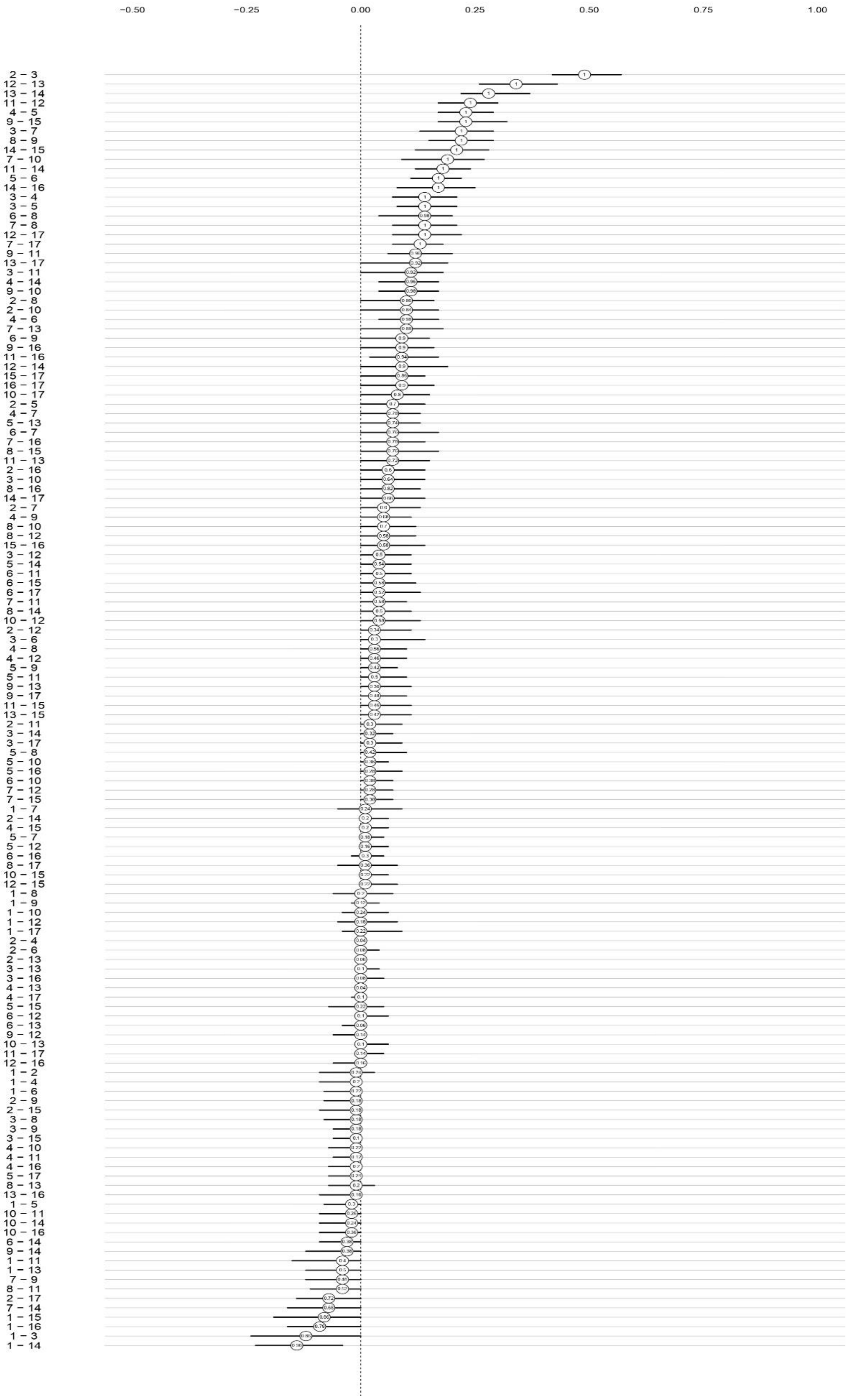

Figure S2. Bootstrapped sampling distribution of the edge weights of the regularized network at post treatment
