# Supplementary material for: Comparing psychological versus pharmacological treatment in emotional disorders: A network analysis
Source: PLoS One. 2024 Apr 3;19(4):e0301675. doi: 10.1371/journal.pone.0301675 (PMC10990220; doi:10.1371/journal.pone.0301675)
Supplement: S10 Fig — (PDF) [file pone.0301675.s010.pdf]

Supplementary Figure S10. Regularized network at twelve months follow-up

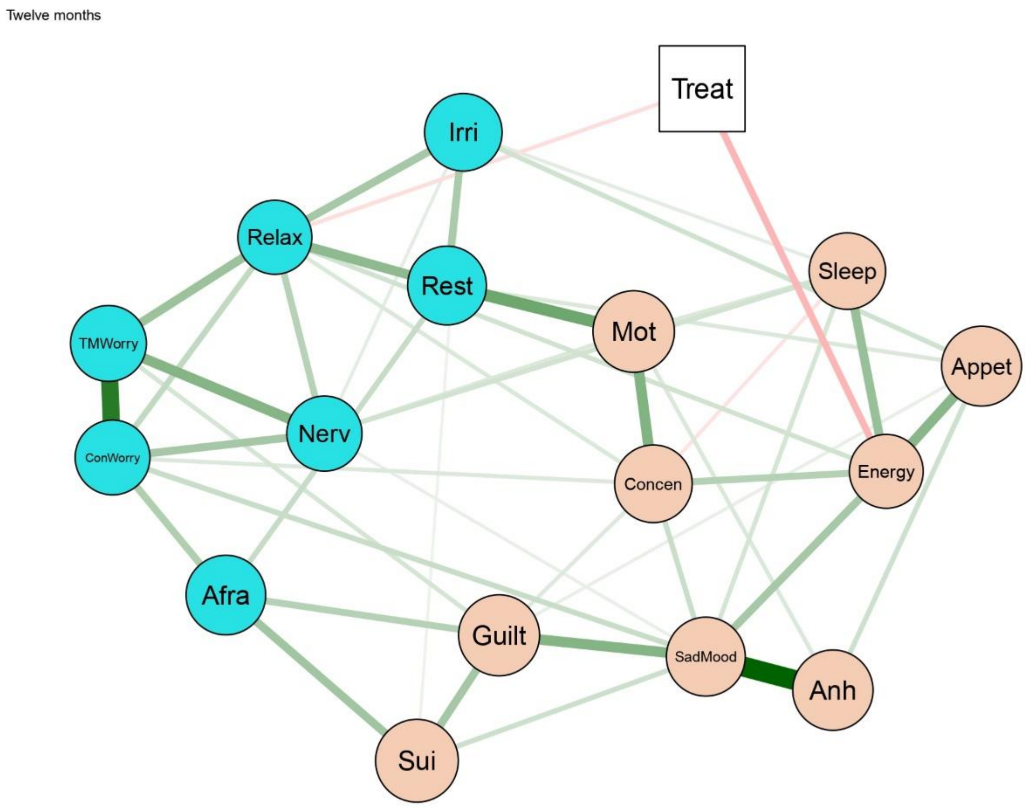

Figure S10. Regularized network at twelve months follow-up using EBIC to select the tuning parameter. The network includes the GAD-7 and PHQ-9 items (circles) and treatment (square). The edges represent the conditional dependence relations among the variables that capture the unique associations among the variables, while controlling for all the other variables in the network. Green edges represent positive associations, red edges represent negative associations, and the thickness and colour saturation of the edge is proportional to the strength of the association. Anh = anhedonia. SadMood = sad mood. Sleep = trouble sleeping. Energy = low energy. Appet = Appetite change. Guilt = Feeling of worthlessness. Concen = concentration difficulties. Mot = psychomotor agitation/retardation. Sui = thoughts of death. Nerv = nervousness or anxiety. ConWorry = uncontrollable worry. TMWorry = worry too much. Relax = trouble relaxing. Rest = restlessness. Irri = irritable. Afra = afraid something will happen.
